# Acute Esophageal Necrosis as a Cause of Pneumomediastinum in a Patient With Diabetic Ketoacidosis

**DOI:** 10.14309/crj.0000000000000568

**Published:** 2021-05-12

**Authors:** Aleksandar Gavric, Samo Plut, Rok Dezman, Gregor Novak

**Affiliations:** 1 Department of Gastroenterology, University Medical Center Ljubljana, Ljubljana, Slovenia; 2 Ljubljana Digestive Endoscopy Research Group (LuDERG), Department of Gastroenterology, University Medical Center Ljubljana, Ljubljana, Slovenia; 3 Institute of Radiology, University Medical Centre Ljubljana, Ljubljana, Slovenia; 4 Medical Faculty Ljubljana, University of Ljubljana, Slovenia

## CASE REPORT

**Figure 1.**
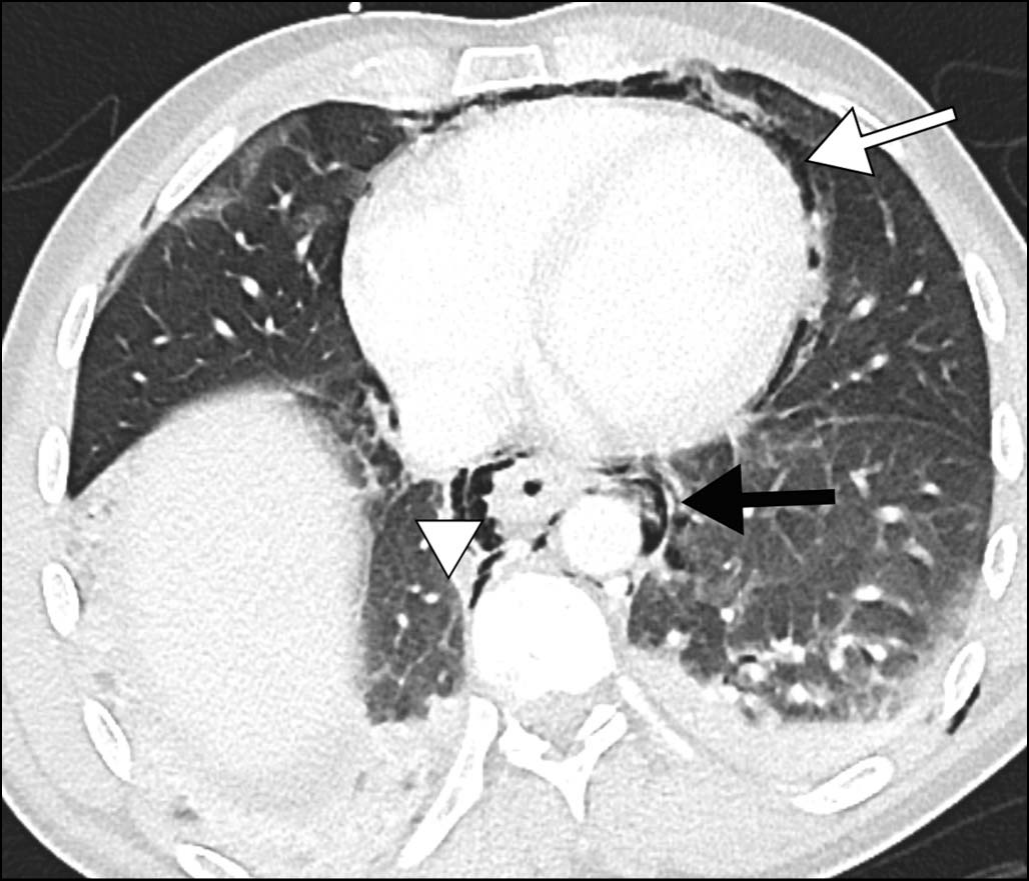
Contrast-enhanced computed tomography showing an extensive pneumomediastinum with pneumopericardium and subcutaneous emphysema of the anterior thoracic wall and neck.

**Figure 2.**
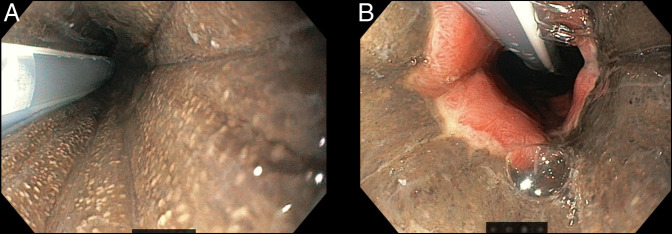
Esophagogastroduodenoscopy revealed a striking diffuse circumferential black mucosal discoloration of the esophagus starting in the upper third (A) and extending to the esophagogastric junction (B).

A 36-year-old man with type 1 diabetes was admitted to intensive care unit because of acute respiratory distress. He had a 3 day history of general malaise, vomiting, and diarrhea. On physical examination, the patient was hypotensive (relative risk 100/60 mm Hg), tachycardic (110/min), dyspnoeic (peripheral oxygen saturation of 93%), and signs of subcutaneous emphysema of the neck. Laboratory results demonstrated metabolic acidosis (pH 7.03), hyperglycemia (667.8 mg/dL), acute kidney injury (creatinine 2.7 mg/dL; baseline 0.86 mg/dL), leukocytosis (32.1 × 10^9^), and increased C-reactive protein (70 mg/L). Hemoglobin was normal (16.5 g/dL). He was intubated shortly after admission. Contrast-enhanced computed tomography (CT) demonstrated an extensive pneumomediastinum with pneumopericardium and subcutaneous emphysema of the anterior thoracic wall and neck (Figure 1). The esophageal wall was diffusely thickened and hypodense. Thoracic CT with ingestion of oral contrast demonstrated no leakage of contrast from the esophagus. Bronchoscopy showed laryngeal edema but no sign of tracheal perforation. Esophagogastroduodenoscopy revealed a striking diffuse circumferential black mucosal discoloration of the esophagus starting in the upper third and extending to the esophagogastric junction (Figure 2). The stomach and duodenum were unremarkable.

Pathohistological analysis of the esophageal changes was not performed because of presumed high risk of biopsy-related perforation. Laboratory results were consistent with diabetic ketoacidosis which was a consequence of noncompliance with insulin. Based on endoscopic appearance, diagnosis of acute esophageal necrosis (AEN) also known as black esophagus syndrome was made. The patient was treated with intravenous antibiotics, insulin, proton pump inhibitors, and parenteral nutrition.

**Figure 3.**
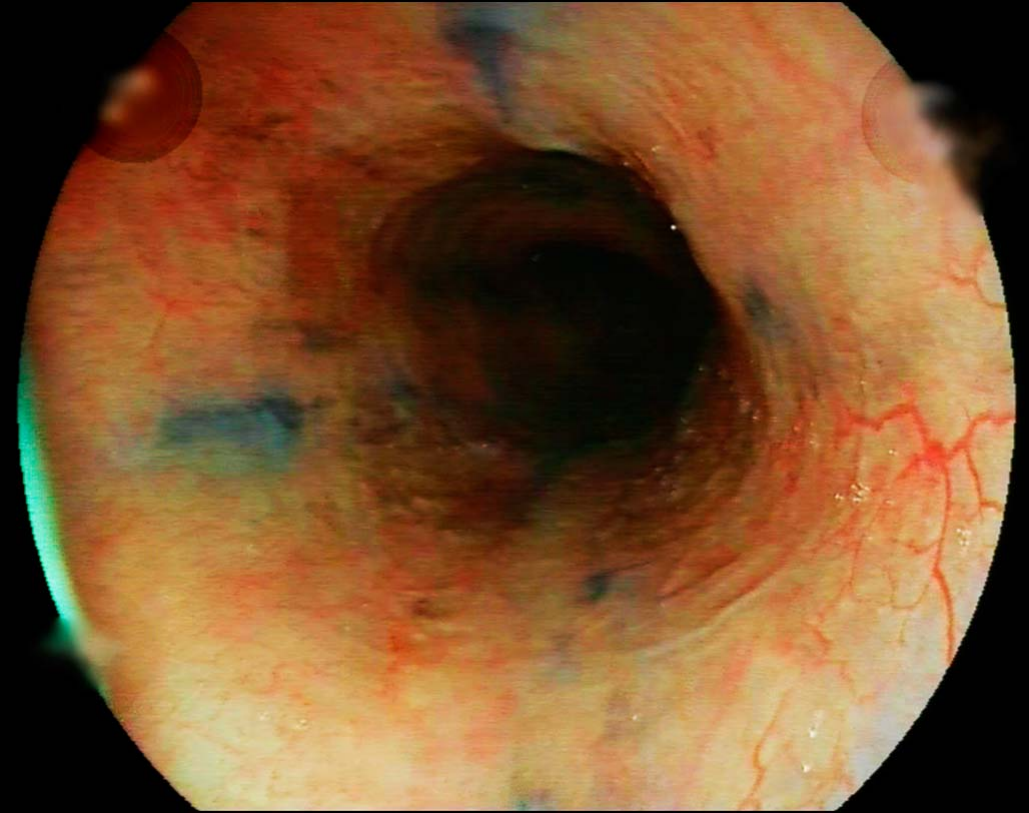
Follow-up endoscopy performed after 10 weeks demonstrated a completely healed esophageal mucosa without strictures.

After 2 days, the CT was repeated showing partial regression of the pneumomediastinum, and the patient was extubated after 3 days. Followup endoscopy performed after 10 weeks demonstrated a completely healed esophageal mucosa without strictures (Figure 3). The patient was discharged home after 3 weeks and was well at an outpatient visit 3 months after discharge.

AEN is a rare clinical entity with high mortality (30%). Surgical intervention is reserved for those who deteriorate despite supportive treatment.^1^ Etiology is not yet entirely explained, but diabetic ketoacidosis has been proposed as one among numerous potential triggers.^1^ The causative mechanism of injury is hypovolemia and ischemia.^1^ The most common clinical presentation (85%) is upper gastrointestinal bleeding, which interestingly was not presented in our patient.^2^ We hypothesize that AEN caused microperforations of the esophageal wall which leads to pneumomediastinum and subcutaneous emphysema in our patient. To best of our knowledge, only 1 case of AEN with pneumomediastinum and subcutaneous emphysema has been published so far.^3^

## DISCLOSURES

Author contributions: All authors contributed equally to the work presented in this article. A. Gavric is article guarantor.

Acknowledgement: The authors thank Sanela Banovic, MD, and Anja Rihtarsic, MD, for providing endoscopy images.

Financial disclosure: None to report.

Informed consent was obtained for this case report.
